# Breast cancer recurrence in relation to mode of detection: implications on personalized surveillance

**DOI:** 10.1007/s10549-024-07475-8

**Published:** 2024-09-10

**Authors:** Hanna Sartor, Oskar Hagberg, Oskar Hemmingsson, Kristina Lång, Charlotta Wadsten

**Affiliations:** 1 Department of Translational Medicine, Diagnostic Radiology, Lund University, Lund, Sweden; 2 Unilabs Breast Unit, Skåne University Hospital, Lund/Malmö, Sweden; 3 Department of Diagnostics and Intervention, Umeå University, Umeå, Sweden; 4 Department of Surgery, Sundsvall Hospital, Sundsvall, Sweden; 5 Dept of Surgery, Sundsvall Hospital, 851 86 Sundsvall, Sweden

**Keywords:** Breast cancer, Locoregional recurrence, Surveillance, Second primary breast cancer, Personalized follow-up

## Abstract

**Purpose:**

The effectiveness of current follow-up guidelines after breast cancer treatment is uncertain. Tailored surveillance based on patient age and tumor characteristics may be more adequate. This study aimed to analyze the frequency of ipsilateral locoregional recurrences (LR) and second primary breast cancers (SP) detected outside of scheduled surveillance and to analyze risk factors associated with these events.

**Methods:**

Patients with surgically treated early-stage breast cancer from the Malmö Diet and Cancer Study (MDCS), 1991–2014 (*n* = 1080), and the Västernorrland region, 2009–2018 (*n* = 1648), were included. Clinical and pathological information on the primary tumor and recurrences was retrieved from medical records. The mode of recurrence detection was defined as detection within (planned) or outside (symptomatic) of scheduled surveillance.

**Results:**

The median follow-up was 6.5 years. Overall, 461 patients experienced a recurrence. The most common initial event was distant metastasis (47%), followed by locoregional recurrence (LR) (22%) and second primary (SP) (18%). 56% of LR and 28% of SP were identified outside of scheduled surveillance. Logistic regression analysis revealed that younger age (under 50 years) (OR 2.57, 95% CI 1.04–6.88), lymph node-positive breast cancer (OR 2.10, 95% CI 1.03–4.39) and breast cancer of the HER2 positive subtype (OR 5.24, 95% CI 1.40–25.90) were correlated with higher odds of detecting a recurrence outside of planned surveillance.

**Conclusion:**

Most recurrent events were detected outside of scheduled surveillance, particularly for locoregional recurrences. Risk-based surveillance, which takes into account patient and tumor characteristics, might be more suitable for specific patient subsets.

**Supplementary Information:**

The online version contains supplementary material available at 10.1007/s10549-024-07475-8.

## Introduction

The prevalence of breast cancer is escalating due to both an increased incidence and improved 5-year survival rates, which surpass 90% in high-income countries [1, 2]. The surveillance of individuals after breast cancer treatment aims to detect curable recurrences, provide psychosocial support, and monitor side effects of completed and ongoing adjuvant treatments [3–5]. However, there is a paucity of evidence-based data to optimally design surveillance programs concerning frequency, modality, and duration of follow-up care in cancer survivors [6]. Observational studies imply that early detection of ipsilateral recurrences or secondary primary breast cancers in the contra-lateral breast confers survival benefits [7, 8]. A meta-analysis involving thirteen studies indicated that survival improves by a hazard ratio of 1.68 (95% confidence interval (CI) 1.48–1.91) in patients with asymptomatic recurrences as compared to symptomatic ones, and it improves by 2.44 (95% CI 1.78–3.35) when the recurrence is identified via mammography rather than a physical examination [7]. Conversely, a 2019 Cochrane review of randomized trials suggested that less intensive follow-up compared to a more rigorous one probably delays the detection of recurrence without altering overall survival [6]. Most local and regional recurrences are detected outside of organized surveillance [9–11], and randomized controlled trials have failed to show that reduced in-hospital follow-up strategies negatively impact either patient-reported outcomes or the early detection of recurrence [4].

The overall risk of loco-regional recurrent breast cancer is low (0.3–0.5% per year after breast conserving therapy) [12], but, to detect a recurrence is of great importance to the affected women. The risk is highest in the first five years, peaking in the second year after treatment and varies based on the patient’s age, features of the initial breast cancer (such as tumor stage, nodal stage, tumor biological subtype), and adjuvant treatments [13–17]. Despite this, no individualized, risk-based follow-up is currently implemented. With increased knowledge, women may be subject to more intense follow-up, but importantly, also less intense follow-up because they are time-consuming and costly for both caregivers and patients.

With the aim of establishing a personalized surveillance system, a comprehensive retrospective analysis of current follow-up practices and patient outcomes is essential. The objective of this study was to examine the frequency of detecting ipsilateral locoregional recurrences (LR) and secondary primary breast cancers (SP) beyond scheduled surveillance, and to analyze risk factors associated with these events in patients suffering from recurring breast cancer.

## Patients and methods

The study population was retrieved from two different cohorts.

### Malmö diet and cancer study (MDCS)

The Malmö Diet and Cancer Study (MDCS) [18, 19] encompassed inhabitants of Malmö from 1991 to 1996, of whom 17,035 were women. The cohort features data on vital status, causes of death, and cancer diagnoses, which are regularly updated through the Swedish Cancer Registry and the Swedish Cause of Death Registry. All women in the cohort diagnosed with breast cancer (from 1991 to 2014) were identified. Women with prevalent breast cancer at baseline (*n* = 576), bilateral breast cancer (*n* = 21), or cancer in situ (*n* = 105) were excluded. Additionally, 16 patients were excluded due to metastatic disease present at the initial breast cancer diagnosis, and 18 patients were excluded due to missing information regarding breast cancer recurrence. A total of 1080 women remained eligible for inclusion after implementing the exclusion criteria.

Information about clinicopathological factors in the MDCS has been described in detail in a previous publication [20]. The status of axillary lymph node involvement was obtained from medical records. Hormone receptor status, based on immunohistochemical (IHC) staining, was extracted from Tissue Microarray (TMA) evaluations from 1991 to 2004 and medical records from 2005 to 2014. The Human Epidermal Growth Factor Receptor 2 (HER2) status was gathered from TMA assessments from 1991 to 2007 and from medical records from 2008 to 2014. Information on IHC proliferation marker Ki67 expression was collected over three periods: from TMA assessments between 1991 and 2004 and 2005–2007, and from medical records from 2008 to 2014. The tumors were then classified into surrogate molecular breast cancer subtypes according to the St. Gallen 2013 guidelines (25). The classifications used: Luminal A-like (ER > 10%, HER2 negative, and Ki67 low), Luminal B-like (ER > 10%, HER2 negative and Ki67 high or Ki67 intermediate and PR < 10%), HER2 + subtype (any ER, PR, Ki67, and HER2 positive), and Triple Negative Breast Cancer (TNBC) (ER < 10%, PR < 10%, HER2 negative, and any Ki67).

The MDCS was conducted according to the Declaration of Helsinki and received approval from the Ethics Review Board in Lund, Sweden (Official Records Nos. 652/2005, 166/2007, and 2014/830) as well as the Swedish Ethical Review Authority (2022-04473-02). In the present study, a subset of women from the MDCS cohort was included, and they provided their informed consent at the baseline.

### Region Västernorrland

Women with breast cancer in the Västernorrland region between 2009 and 2018 were identified from The Swedish National Breast Cancer Quality Registry (*n* = 2090). This registry has had national coverage since 2007 and includes all incident breast cancers in Sweden, with reported completeness close to 100% [21]. Women initially diagnosed with metastatic disease (*n* = 79), those who did not undergo surgery due to other reasons (*n* = 81), women with synchronous bilateral cancer (*n* = 29), and women with cancer in situ (*n* = 253) were excluded. These exclusions left 1648 women eligible for the analyses.

The age at diagnosis, nodal status, breast surgery, hormone receptor status, HER2 status, and Ki67 of the index breast cancer were obtained from medical records. The Swedish Ethical Review Authority approved the data collection for this study (2020-02165 and 2024-00428-02), waiving the need to secure informed consent.

### Follow-up routines and outcomes

In MDCS, the recommended follow-up after breast cancer treatment included annual mammography for ten years for patients who underwent breast-conserving surgery (BCS). Patients treated with mastectomy were incorporated into the standard screening program. The schedule for in-hospital physical examinations varied during the study period, primarily comprising annual visits for the first three years and capped off with a final visit at five years post-diagnosis.

In Västernorrland, recommended follow-up included annual bilateral mammography for five years for all patients, regardless of whether BCS or mastectomy had been performed. Further, patients receiving adjuvant chemotherapy were also offered annual in-hospital physical examinations for a duration of 5 years.

Recurrent events were defined in accordance with the consensus on event definitions for classifying recurrence in breast cancer, as published in 2014 [22].

The time of follow-up initiated at the date of diagnosis and concluded at the date of the first relapse either in the ipsilateral breast, scar, chest wall, or ipsilateral regional lymph nodes (LR) or in the instance of a second primary breast cancer in the contralateral breast (SP) or distant metastasis including contralateral regional lymphnodes as the first event (DM). Patients experiencing synchronous bilateral locoregional events were designated as LR, and those with LR or SP and synchronous DM as the initial event were classified as LR + DM. HS (MDSC) and CW (Västernorrland) retrospectively reviewed the mode of recurrence detection (planned or symptomatic) within the scope of this present study. Recurrent events were deemed symptomatic when identified at a patient-oriented visit or breast imaging outside of scheduled surveillance.

### Statistics

To investigate whether the variables - age at diagnosis, nodal stage, subtype, or type of surgery - were associated with a larger proportion of recurrences detected outside of scheduled surveillance, a multivariable logistic regression model was applied. This analysis included LR, SP and LR + DM but excluded patients with DM only because distant metastases are typically detected symptomatically and scheduled surveillance for detecting DM is not recommended. A composite model that used the type of recurrence (LR, SP and LR + DM) as a covariate was applied. Hence the result should be interpreted *conditional* on getting LR, SP or LR + DM. Given that this study focused on variables associated with the risk of symptomatic detection, no formal time-to-event analysis of the risk of recurrence was performed. Only summary measures of the size of the four groups - LR, SP, LR + DM, and DM - and the time from diagnosis to recurrence were provided.

## Results

**Fig. 1:**
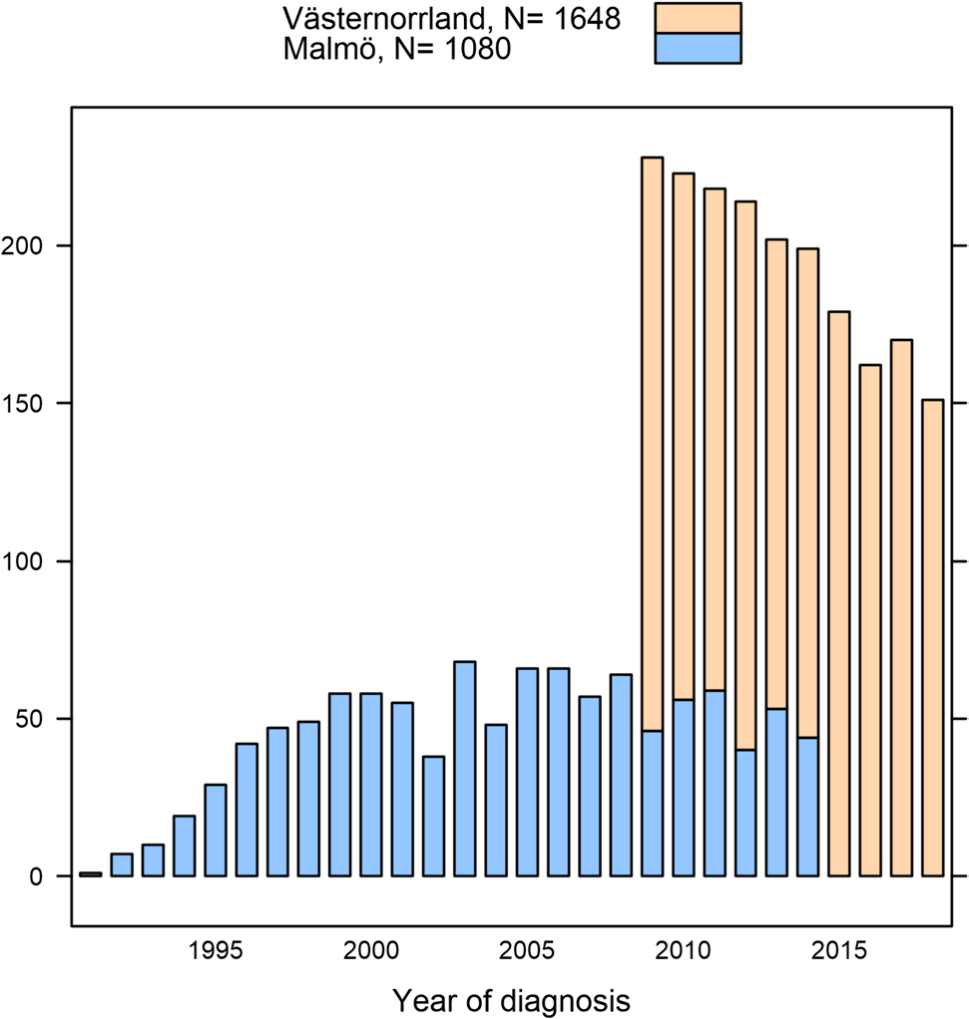
Year of breast cancer diagnosis in the Malmö Diet and Cancer Study and region Västernorrland

The years of breast cancer diagnosis for the two separate cohorts are displayed in Fig. 1. The women in the MDCS cohort received their breast cancer diagnoses during an earlier period compared to the women in the Västernorrland cohort, with a 6-year overlap. The median age at the time of breast cancer diagnosis was 66 years (interquartile range, IQR: 59–72), and the median follow-up period was 6.5 years (IQR: 3.8–10.0) (Table 1).

**Table 1:** Patient characteristics

	Total	Only recurred	
N (%)		All	Except DM
*N Total*	*2728*	*461*	*243*
Age at diagnosis (years)			
Mean (SD)	65.1 (11.2)	64.1 (10.8)	62.8 (10.5)
Median (IQR)	66 (59–72)	64 (57–72)	63 (56–69)
Range	23–97	29–92	29–88
N-stage			
N0	1761(70.2)	232(53.3)	161(72.2)
N+	746(29.8)	203(46.7)	62(27.8)
< NA>	221	26	20
Subtype			
Luminal A-like	1251(52.0)	144(38.4)	95(49.2)
Luminal B-like	638(26.5)	126(33.6)	45(23.3)
HER2+	280(11.6)	45(12.0)	19(9.8)
Triple-negative	238(9.9)	60(16.0)	34(17.6)
< NA>	321	86	50
Breast surgery			
BCS	1609(60.8)	205(45.4)	135(56.7)
Mastectomy	1037(39.2)	247(54.6)	103(43.3)
< NA>	82	9	5

*N* Numbers, *DM* Distant metastasis, *SD* Standard deviation, *IQR* Interquartile range, *N*-stage Nodal stage, *NA* not available, *HER2* Human epidermal growth factor receptor 2, *BCS* Breast conserving surgery

In total, 461 patients experienced a recurring event. The median time to recurrence was 4.31 years (IQR 2.05–8.19 years) (Suppl Table 1). The distribution of the first relapse in the two distinct cohorts is illustrated in Table 2. The most common initial event was DM (47%), followed by LR (22%) and SP (18%).

**Table 2:** Distribution of type of first recurrence in the Malmö Diet and Cancer Study and in region Västernorrland. Originally, recurrences were divided into seven categories, LR (local recurrence), SP (second primary), DM (distant metastasis), LR + SP, LR + DM, SP + DM, and LR + SP + DM. In the analyses in this study, these are in turn grouped into four larger categories as shown in the first column in bold

*n* (%)	Malmö	Västernorrland	Total
Total no. of Recurrences	262	199	461
LR	**60 (23)**	**43 (22)**	**103 (22)**
LR	58 (22)	41 (21)	99 (21)
LR + SP	2 (1)	2 (1)	4 (1)
SP	**48 (18)**	**33 (17)**	**81 (18)**
SP	48 (18)	33 (17)	81 (18)
LR + DM	**36 (14)**	**23 (12)**	**59 (13)**
LR + DM	21 (8)	21 (11)	42 (9)
SP + DM	14 (5)	1 (1)	15 (3)
LR + SP + DM	1 (0)	1 (1)	2 (0)
DM	**118 (45)**	**100 (50)**	**218 (47)**
DM	118 (45)	100 (50)	218 (47)

*LR* Local recurrence, *SP* Second primary, *DM* Distant metastasis, *No* Numbers

Table 3 illustrates the distribution of recurrences categorized by age at the time of primary breast cancer diagnosis, tumor subtype, nodal stage, and type of breast surgery. DM as the initial event was most prevalent in patients with Luminal B-like (81 of 126, 64.3%) and HER2 positive tumors (26 of 45, 57.8%), whereas it was least prevalent in patients with Luminal A-like tumors (49 of 144, 34.0%). Moreover, DM as the initial event was notably common in patients with lymph node-positive primary breast cancer (141 of 203, 69.5%) and in patients who had undergone a mastectomy, as opposed to those who had BCS (144 of 247, 58.3%).

**Table 3:** Type of recurrence by age, N-stage, subtype, and surgery, row percentage

*n* (%)	LR	SP	LR + DM	DM	Total
Total no. of Recurrences	103 (22.3)	81 (17.6)	59 (12.8)	218 (47.3)	461
Age at diagnosis (Years)					
Mean (SD)	63.2 (10.7)	62.0 (9.3)	63.2 (11.7)	65.6 (11.0)	64.1 (10.8)
Median (IQR)	63 (56–70)	63 (57–67)	64 (56–71)	66 (58–74)	64 (57–72)
Range	38–88	29–82	32–85	36–92	29–92
N-stage					
N0	72 (31.0)	64 (27.6)	25 (10.8)	71 (30.6)	232
N+	22 (10.8)	14 (6.9)	26 (12.8)	141 (69.5)	203
< NA>	9	3	8	6	26
Subtype					
Luminal A-like	45 (31.2)	37 (25.7)	13 (9.0)	49 (34.0)	144
Luminal B-like	14 (11.1)	14 (11.1)	17 (13.5)	81 (64.3)	126
HER2+	9 (20.0)	4 (8.9)	6 (13.3)	26 (57.8)	45
Triple-negative	22 (36.7)	5 (8.3)	7 (11.7)	26 (43.3)	60
< NA>	13	21	16	36	86
Type of breast surgery					
BCS	65(31.7)	46(22.4)	24(11.7)	70(34.1)	205
Mastectomy	36(14.6)	35(14.2)	32(13.0)	144(58.3)	247
< NA>	2	0	3	4	9

*N-stage* Nodal stage, *LR* Local recurrence, *SP* Second primary, *DM* Distant metastasis, *No *Numbers, *SD* standard deviation, *IQR* interquartile range, *NA* Not available, *HER2* Human Epidermal growth factor receptor 2, *BCS* Breast conserving surgery

### Risk of recurrence detected outside of scheduled surveillance

Overall, 298 out of 417 (71.5%) first recurrent events were detected outside of scheduled surveillance (Table 4). Of the ipsilateral LR, 53 out of 95 (56%) were detected outside of scheduled surveillance, while 54 out of 75 (72%) SPs in the contralateral breast were identified during scheduled mammography appointments. Almost all DMs were detected due to symptoms, with 183 out of 193 (95%) cases.

**Table 4:** Detection method for different types of recurrence

*n* (%)	LR	SP	LR + DM	DM
Planned	42 (44)	54 (72)	13 (24)	10 (5)
Symptomatic	53 (56)	21 (28)	41 (76)	183 (95)
Missing	8	6	5	25
Total	103	81	59	218

*LR* Local recurrence, *SP* Second primary, *DM* Distant metastasis

### Recurrence detection in relation to index breast cancer features

After excluding patients whose first event was DM, a logistic regression analysis was executed to identify risk factors for the detection of symptomatic LR and SP. This analysis revealed that young age (< 50 years) (OR 2.57, 95% CI 1.04–6.88), lymph node-positive breast cancer (OR 2.10, 95% CI 1.03–4.39), and HER2-positive subtype breast cancer (OR 5.24, 95% CI 1.40–25.90) were associated with an increased likelihood of recurrence detection outside of scheduled surveillance (Table 5).

**Table 5:** Variables associated with recurrence detected outside of scheduled surveillance. A logistic regression model was employed to adjust for the three different types of recurrence (Loco-regional (LR), second primary breast cancer (SP), or synchronous LR/SP with distant metastasis, LR + DM) and the odds ratio jointly for these types was estimated for a number of categorical variables

		Recurrence detected outside surveillance	%	Odds Ratio (95% CI)
Age at diagnosis	51–97	96/197	48.7	1
	23–50	19/27	70.4	2.57 (1.04–6.88)
N stage	N0	66/150	44.0	1
	N+	37/54	68.5	2.10 (1.03–4.39)
Subtype	Luminal A-like	39/91	42.9	1
	Luminal B-like	25/44	56.8	1.29 (0.56–2.98)
	HER2+	13/16	81.2	5.24 (1.40–25.90)
	Triple-negative	18/30	60.0	1.45 (0.59–3.66)
Type of surgery	BCS	61/130	46.9	1
	Mastectomy	51/89	57.3	1.42 (0.79–2.58)

*CI* Confidence interval, *N stage* Nodal stage, *HER2* Human epidermal growth factor receptor 2, *BCS* Breast conserving surgery

## Discussion

This study corroborates previous research, stating that a majority of all recurrences (71.5%), which includes distant metastasis, and 56.0% of ipsilateral locoregional recurrences are discovered outside of scheduled surveillance (9–11, 22–24). There were fewer overall recurrences in Västernorrland, reflecting the more contemporary period of breast cancer diagnoses in this population. However, the proportions of LR, SP and DM were notably similar between the two cohorts. Importantly, the probability of detecting a recurrence outside of surveillance was higher among younger patients, patients with lymph node-positive breast cancer, and cases of the HER2-positive breast cancer subtype.

### Locoregional recurrences

The effectiveness of current surveillance programs has been previously examined, yielding mixed results. The reported proportion of ipsilateral recurrent events identified through scheduled annual mammography varies between 8% and 51% [9, 11, 23–25]. In contrast, recurrence detected during routine physical examinations is rare [9, 26, 27].

Governmental institutions and medical societies typically recommend annual bilateral mammography following breast-conservative surgery, while most suggest only contralateral mammography for patients who underwent a mastectomy [3, 4, 28–30]. Routine ultrasound, however, is not advised [28]. The benefits of digital breast tomography and magnetic resonance imaging (MRI) in routine surveillance remain unclear. MRI is suggested to be more beneficial for young women (i.e., those under 50 years) or women at higher risk of recurrence, such as those with dense breasts or a first-degree family history [29, 30]. The effectiveness of more frequent mammography intervals remains unconfirmed [31–33].

Advancements in breast cancer management have led to a decline in the overall rate of locoregional recurrences [12, 14, 16]. Currently, the estimated LR risk following breast-conserving surgery (BCS) coupled with whole breast radiotherapy is reported to be 0.3–0.5% per year. The annual risk of SP breast cancer is approximately 0.4% per year [12, 34]. This prompts the question of whether follow-up could be de-escalated, at least for certain subsets of patients. The preliminary results from the Mammo-50 trial in the UK were recently presented, where 5000 female patients aged over 50 who were relapse-free following 3 years of standard surveillance were randomized between less frequent mammograms and continued annual mammograms. After a median follow-up of 5.7 years, there was no difference in survival, recurrence rates, or quality of life [35]. This data suggests that less intensive surveillance is feasible for patients with a predicted low risk of recurrence.

### Second primary

The effectiveness of mammography surveillance seems to be more significant for the detection of SP breast cancer, a result that aligns with this study [9, 11, 25, 36]. SP in the opposite breast often gets detected at an earlier stage compared to the initial breast cancer [36]. Nonetheless, the survival benefit of early SP detection remains uncertain due to the competing risk posed by the primary breast cancer [36].

### Personalized risk-based surveillance

In the era of personalized precision medicine, specifically pertaining to breast cancer treatment, surveillance recommendations remain generalized. Despite the variations in recurrence risk by age, tumor subtype, and nodal stage [14–17], current surveillance methods lack individualization. Furthermore, it is not well-examined whether early detection of recurrences is more beneficial, contingent upon patient and tumor characteristics. In other words, it remains to be seen whether more intensive surveillance would be advantageous for certain individuals. A recent observational study suggested that asymptomatic imaging, compared to symptomatic presentation of DM, was associated with a lower risk of death for triple-negative and HER2-positive breast cancer. However, it did not make a significant difference for the luminal-like breast cancers [37]. Whether this also applies for in-breast recurrences is unclear. In the present study, young age, lymph node-positive breast cancer, and HER2-positive subtype were associated with symptomatic detection of recurrences. This possibly indicates that these patients could benefit from more intensive surveillance, including supplemental imaging, during the early follow-up years. To better assist personalized follow-up strategies, the INFLUENCE 1.0 nomogram was developed in the Netherlands in 2015. This nomogram underwent further development in 2021 (INFLUENCE 2.0) and 2024 (INFLUENCE 3.0) [15, 38, 39]. It estimates an individualized risk of recurrence based on age, mode of detection, type of surgery, grade, nodal stage, multifocality, hormone receptor status, HER2 status, and adjuvant treatments.

This tool ought to be advantageous for personalized follow-up regimes. With local recurrence rates decreasing, it presents an opportunity to lessen surveillance for patients with an exceptionally low risk of recurrence. Further refinement in the accuracy of risk prediction models, validated across a diverse set of breast cancer survivors, would be beneficial for the development of risk-based imaging surveillance. High breast density at the time of primary breast cancer diagnosis correlates with an increased risk of recurrence and could, therefore, be incorporated into a model for tailored surveillance in a prospective study design [40].

Several methodological issues require attention. Firstly, in the current study, data pertaining to follow-up and detection methods of recurrences were gathered retrospectively from a large combined study population spanning two distinct geographical areas. Covering a period from 1991 to 2019 means that the study displays both strengths and limitations–treatments have progressed, and surveillance routines have varied over this period and between the two cohorts, potentially impacting the interpretation of the results. The surveillance routines in the MDCS cohort and the Västernorrland cohort varied in both interval and duration. In the MDCS, surveillance interval was stratified by surgery while all breast cancer patients in Västernorrland underwent yearly mammography. Clinical examinations have been de-escalated over time in both cohorts. Secondly, one limitation is the retrospective gathering of data, which requires the retrospective assessment of medical journals and radiologic reports to determine if mammography was performed within or outside scheduled surveillance. Additionally, information on the adherence to scheduled surveillance among all patients in the study population was not available.

In conclusion, even though the risk of recurrence varies with patient age and tumor characteristics, surveillance recommendations remain generalized. Most recurrences are detected outside of scheduled surveillance. This study indicates that young age, lymph node-positive breast cancer, and breast cancer of the HER2-positive subtype may call for more intensive surveillance.

## Supplementary Information

Below is the link to the electronic supplementary material.

Supplementary material 1 (DOCX 14.0 kb)

## Data Availability

No datasets were generated or analysed during the current study.
