# Supplementary material for: Breast cancer recurrence in relation to mode of detection: implications on personalized surveillance
Source: Breast Cancer Res Treat. 2024 Sep 10;209(1):85–92. doi: 10.1007/s10549-024-07475-8 (PMC11785654; doi:10.1007/s10549-024-07475-8)
Supplement: Supplementary file 1 — Supplementary material 1 (DOCX 14.0 kb) [file 10549_2024_7475_MOESM1_ESM.docx]

*Supplemental Table 1: Median years to recurrence by type.*

|  | Median  (years) | IQR |
| --- | --- | --- |
| LR | 5.37 | 2.32-9.43 |
| SP | 5.43 | 2.99-9.12 |
| LR+DM | 4.28 | 1.94-8.24 |
| DM | 3.61 | 1.80-7.03 |
| Total | 4.31 | 2.05-8.19 |

IQR = interquartile range, LR = local recurrence,

SP = second primary, DM = distant metastasis
